# The system and characteristics of teacher beliefs of preservice physical education teachers in China: a qualitative approach

**DOI:** 10.3389/fpsyg.2025.1666929

**Published:** 2025-12-29

**Authors:** Lin Li

**Affiliations:** School of Sports Management and Communication, Capital University of Physical Education and Sports, Beijing, China

**Keywords:** physical education, physical education teaching education, preservice teacher, system, teacher beliefs

## Abstract

A qualitative study based on grounded theory was carried out to analyse the system of teacher beliefs of preservice physical education teachers (PPETs) in China. The study aimed to understand the components and characteristics of PPETs’ beliefs in current China and a total of 25 participants from three Physical Education Teaching Education (PETE) programs in three different types of universities participated in this study. The data were collected through semi-structured interviews. There were three core categories identified, (1) professional cognition and (2) emotional experience and (3) behavioural disposition. Professional cognition comprises four components including curriculum comprehension, understanding of PE learning, teacher cognition and cognition of the teaching process. Emotional experience comprises three components including initial emotions, emotional development and emotional sublimation. Behavioural disposition comprises three components including behavioural experience of the vocation, behavioural choice of the vocation and future plans. It is also found the system of PPETs’ beliefs is dynamic and can be divided into different phases. The findings contribute to the theoretical understanding of PPETs’ beliefs and some suggestions for teacher educators are provided.

## Introduction

Physical education (PE) plays a vital role in promoting physical health, mental well-being, and social skills among students. Whether PE could fully play the role depends on PE teacher beliefs, which is defined as “individual’s judgement of the truth or falsity of a proposition” (Pajares, 1992), which could significantly influence their instructional practices (Pajares, 1992; Borg, 2001). Teacher beliefs are foundational to the teachers’ instructional strategies, classroom interactions even long-term career trajectories. For PE teachers, especially PPETs, teacher beliefs play an essential role in cultivating students’ physical health, improving motor skills and promoting the concept of lifelong health (Kulinna et al., 2010). However, current research on the belief system of PPETs remains relatively limited (Tsangaridou, 2012; Richards et al., 2014). Existing studies predominantly concentrate on the belief systems of in-service teachers, leaving teacher beliefs of PPETs underexplored. What’s more, PPETs are in a transitional phase, evolving from students to educators, during which their system of teacher beliefs is still in the process of formation. Consequently, investigating the belief system of this group is of significant importance.

Teacher beliefs are defined as the firm convictions they hold regarding teaching, learning, disciplinary knowledge, and their own roles (Pajares, 1992; Borg, 2001). For instance, Borg (2001) posits that beliefs encompass perspectives on language, learning, curriculum, and more. In the field of PE, these beliefs further differentiate into cognitions about PE teaching goals and teaching methods (Leonardo et al., 2022). This indicates that there are differences in the components of teacher beliefs among different subjects.

This study aims to explore the system of PPETs’ beliefs by identifying their core components and examining the interrelationships among them. The primary goal is to contribute to a theoretical system of teacher beliefs of PPETs. Ultimately, the findings are designed to provide a foundation for PE educators and for PETE programs.

### The definition of teacher beliefs

Since the 1980s, with the continuous deepening research in the cognitive field, the focus from teachers’ behaviours has been shifted to the knowledge and beliefs behind their behaviours. Beliefs are individual mental construction, which determine decisions and actions. However, it is also a “bewildering array of terms” and beliefs travel in disguise and often under alias-attitudes, values, rules of practice (Pajares, 1992). In the process of sorting out the concept of teacher beliefs, it is found that there is a consensus that teacher beliefs belong to a cognitive domain held by individuals. However, some scholars believe that teacher beliefs also involve affective and behavioural components in addition to cognitive components. Rokeach (1968) argued that teacher beliefs contain an emotional component that triggers emotions. Abelson (1979) also believed that teacher beliefs include emotion and assessment, which had been affirmed by the academic community. Nespor (1987) suggested that beliefs have stronger affective components than knowledge.

### The research of teacher beliefs of PPETs

Research on PE teacher beliefs have primarily centered on domains tied to instructional outcomes, such as teaching efficacy (Hand, 2014; Pendergast et al., 2011), perceived professional roles (McCullick et al., 2012; Rachele et al., 2016), pre-service preparation (Chróinín and O’Sullivan, 2016), and intended curriculum outcomes (Adamakis and Zounhia, 2015). These studies have advanced understanding of how PE teachers’ cognitions influence classroom behaviours, from activity adaptation to student engagement management. However, a critical gap persists: the systematic exploration of teacher beliefs of PPETs remains underdeveloped.

Existing research on PPETs’ beliefs has focused disproportionately on influencing factors and the beliefs about the PE profession. Interpersonal relationships, for example, have been identified as a key domain: Curtner-Smith et al. (2008) highlighted the role of PE teachers and coaches during secondary education, while Dodds and Placek (1991) and Lawson (1983) noted familial influences, with González-Calvo et al. (2021) demonstrating that family members’ sports enthusiasm enhances PPETs’ passion for physical activity. In addition, peer interactions also play a part. Ferry (2018) observed that mutual support among university peers deepens PPETs’ understanding of teaching and learning. Teaching practice, including student feedback (Korthagen and Vasalos, 2005; Harvey et al., 2013) and educational internships, further shapes beliefs. For PPETs, the research of their belief was paid attention to PE profession and found the sense of calling and value of PE profession (Keating et al., 2020).

Notably, these studies focus on what influences PPETs’ beliefs but not how these beliefs are structured. This lack of clarity limits our ability to theoretically depict PPETs’ belief system and to develop targeted interventions to strengthen their professional identity.

### Research gaps and research questions

A systematic review of the existing literature reveals that while there is a consensus regarding the significance of teacher beliefs and their core cognitive nature, the understanding of their internal structure remains an area requiring further scholarly dialogue. Specifically, the current body of research exhibits three interrelated limitations.

First, there is a notable imbalance in the exploration of the internal constituents of teacher beliefs. Scholars generally agree that teacher beliefs constitute a complex, multi-dimensional systemic structure (Pajares, 1992; Abelson, 1979; Rokeach, 1968). However, prevailing research demonstrates a tendency to be predominantly focused on cognitive aspects at the expense of other dimensions. Although studies have extensively explored its role as a cognitive framework, some scholars, through theoretical deliberation, have suggested that beliefs are not only about pure cognitive components but also about value judgments, such as those related to teaching self-confidence or efficacy (Nespor, 1987; Pajares, 1992). However, the problem lies in the fact that scholarly discussion remains vague and fragmented concerning the specific facets of these important non-cognitive dimensions and how they interact with cognitive components to form a coherent belief system. Secondly, this conceptual ambiguity directly contributes to a static research perspective. The majority of studies strive to depict static snapshots of belief components at a specific point in time, often relying on cross-sectional data. In essence, this paradigm treats the different aspects of beliefs as relatively isolated and fixed units of analysis. Consequently, it fails to capture teacher beliefs as a dynamic system interacting with the real-world complexities of the teaching context, and it cannot reveal the ongoing negotiation and evolutionary trajectory of its internal components. Thirdly, current studies primarily use quantitative scales (Fan et al., 2018; Keating et al., 2020), which risk oversimplifying the complexity of beliefs. Qualitative methods, by contrast, offer nuanced insights into the lived experiences and dynamic processes through which PPETs construct their belief system.

Therefore, this research aims to move beyond the predominant focus on cognitive elements and static descriptions of beliefs as a whole. Instead, it seeks to investigate the richer, internal dimensions of teacher beliefs and their dynamic interplay.

### The significance of the study

Based on the relevant research, this study utilized bottom-up qualitative analysis to explore the system of PPETs’ beliefs. The study also analysed and explored the characteristics of the formation of teacher beliefs. By centering PPETs’ voices through interviews and reflective journal, this research will: (1) identify the core components of their belief systems (2) uncover how these components interact and evolve.

The findings will advance theoretical understanding of PPETs beliefs providing a framework for future research. Practically, they will inform teacher educators about strategies to nurture positive and resilient system of teacher beliefs of PPETs, ultimately enhancing the quality of PETE programs.

## Methodology

### Research positionality and research design

In qualitative research, it is important to maintain transparency regarding an epistemological stance to ensure that the researchers’ influence on the research outcomes is clear. This study adopts the epistemological stance of constructivist grounded theory (Charmaz, 2006). This perspective posits that theory is neither an objective entity discovered nor a purely subjective creation of the researcher. Instead, it is co-constructed through interactions between the researcher and participants, as well as through the processes of data collection and analysis. Grounded theory was selected as an appropriate qualitative methodology to achieve the study’s aim of generating new theory because grounded theory is based upon a theoretical framework of symbolic interactionism (Corbin and Strauss, 2008). Grounded theory is both a process and product of research and it is an appropriate endeavour for studies that seek to develop new knowledge about participants’ experience (Corbin and Strauss, 2008), which is suitable for the study focusing on the experience of PPETs. Therefore, based on the stance, our understanding of teacher beliefs of PPETs is as follows: it is not a pre-existing, fixed psychological structure that can be simply measured, but rather a dynamic, contextualized system of meaning that is continuously shaped and revised through individual experiences. Therefore, the core objective of this study is to generate a theoretical system of PPETs’ beliefs, trying to explore the dynamic formation process and the inherent complex elements of this system within the specific context of a 4-year PETE programs.

### Data collection and theoretical sampling

The theoretical sampling process in this study was dynamic and iterative, driven by the emergent concepts and categories from the ongoing data collection and analysis (Charmaz, 2006; Corbin and Strauss, 2008). It commenced with an initial, purposive sampling of PPETs from a sport university where the author is affiliated.

The initial analysis of interviews from this first group revealed a prominent theme: these PPETs heavily emphasized the acquisition and mastery of sports skills and techniques, which they perceived as the core of their professional identity. Notably, a compelling concept emerged when several participants expressed a belief that their competence as future PE teachers was not different from those from other types of institutions. This notion of “professional competence” became a critical analytic point. It prompted us to question: If they feel equivalent, what might be the distinct characteristics of PPETs from other type of institutional backgrounds? This led to the first theoretical sampling decision. Guided by the that emergent concept as a potential differentiator of teacher beliefs, we purposefully sought a second group of participants from a normal university. In the same way, we selected participants from a comprehensive university, which is for all kinds of future professionals. The theoretical rationale was to explore how an institutional context that blends both disciplinary (sports science) and general teacher education might shape a hybrid or distinct set of beliefs, thereby helping to saturate the properties of our categories related to institutional influence and professional identity formation.

This interviewing process continued until theoretical saturation was reached for categories concerning the development of PPETs beliefs, meaning that new data no longer yielded new categories (Charmaz, 2006; Corbin and Strauss, 2008).

### Participants

The interviewees of this study were students majoring in PE. The sampling of the interviewees was carried out in a manner that began with convenience sampling and gradually incorporated theoretical sampling. Interviews were conducted with senior students majoring in PE from one sport university, one normal university, and one comprehensive university, totalling 25 individuals. In China, PETE programs are offered in three different types of colleges and universities: (a) kinesiology/sport institutes and universities, which aim to prepare professionals for the fields of sports and physical educations; (b) normal colleges and universities, focusing on PE teacher preparation; and (c) comprehensive universities for all kinds of future professionals concerning sports, physical education, fitness, and recreation (Yang et al., 2023).

The participant inclusion criteria were: (Abelson, 1979) being a senior-year student in the PETE programs who had completed the teaching practicum, and (Adamakis and Zounhia, 2015) expressing a commitment to pursuing a career as a school PE teacher. In order to explore PPETs beliefs during the 4-year college life, during interviews, we employed profoundly open-ended questions to encourage participants to articulate their experiences in their own language and narratives.

Guided by the theoretical perspective that teacher beliefs influence instructional practices (Pajares, 1992), this study’s semi-structured interviews were designed to further explore the specific strategies employed by PPETs when they discussed their experiences during the teaching practicum and addressed relevant challenges in teaching contexts. In addition, informed by Rokeach’s (1968) theory of belief hierarchies, the researcher reflected during the coding process on whether a certain category of beliefs held a more central position at different time points. Questions include (1) How would you describe the role of a PE teacher? (2) In your view, what constitutes an outstanding PE teacher? (3) What are the essential elements of a successful PE lesson? (4) Reflecting on your 4-year university experience, how has your understanding of PE teaching evolved? (Table 1).

**Table 1:** Description of participants.

Serial number	Participant	Gender	Age	Type of university	Specialization	Interview format
1	CXD	F	21	Professional sport university	Physical education	Online interview; Face-to-face interview
2	LZY	F	22	Professional sport university	Physical education	Online interview; Face-to-face interview
3	PTG	M	22	Professional sport university	Physical education	Online interview; Face-to-face interview
4	LHW	F	21	Professional sport university	Physical education	Online interview; Face-to-face interview
5	LJ	F	22	Professional sport university	Physical education	Online interview; Face-to-face interview
6	XJL	F	22	Professional sport university	Physical education	Online interview; Face-to-face interview
7	CA	F	21	Professional sport university	Physical education	Online interview; Face-to-face interview
8	CYQ	M	22	Professional sport university	Physical education	Online interview; Face-to-face interview
9	DX	F	22	Professional sport university	Physical education	Online interview; Face-to-face interview
10	LCJ	M	22	Professional sport university	Physical education	Online interview; Face-to-face interview
11	MJZ	F	22	Professional sport university	Physical education	Online interview; Face-to-face interview
12	WF	M	23	Normal university	Physical education	Online interview
13	XHF	M	22	Normal university	Physical education	Online interview
14	YL	M	21	Normal university	Physical education	Online interview
15	ZL	F	22	Normal university	Physical education	Online interview
16	ZXR	F	22	Normal university	Physical education	Online interview
17	ZWJ	M	23	Normal university	Physical education	Online interview
18	ZYZ	M	21	Normal university	Physical education	Online interview
19	ZCK	M	23	Normal university	Physical education	Online interview
20	YD	M	22	Comprehensive university	Physical education	Face-to-face interview
21	YBQ	M	23	Comprehensive university	Physical education	Face-to-face interview
22	ZXY	M	21	Comprehensive university	Physical education	Face-to-face interview
23	YWY	M	23	Comprehensive university	Physical education	Face-to-face interview
24	SZS	M	22	Comprehensive university	Physical education	Face-to-face interview
25	SGH	M	22	Comprehensive university	Physical education	Face-to-face interview

### Data analysis

Prior to analysis, all audio recordings of the interviews were transcribed verbatim. Our perspective during analysis was interpretive in nature. Data analysis followed the systematic, iterative procedures of the grounded theory approach as detailed by Corbin and Strauss (2008). Data collection and analysis occurred concurrently, allowing for the constant comparison of data and the emergent theory to guide subsequent sampling and questioning. Regarding theoretical saturation as the determinant of the endpoint for selective coding in grounded theory, the failure to identify new categories through textual analysis indicates that theoretical saturation has been achieved. During the processing of raw data, textual materials from two interview participants were reserved specifically for validation at this stage. If no new categories or concepts emerge during the validation process, the theoretical model is deemed to have reached saturation. The analysis involved the following key stages.

#### Open coding

Open coding is the process of classifying, comparing, and labelling raw interview data, with the aim of conceptualizing and abstracting the raw material by discarding existing research findings and removing less frequent concepts, and developing concepts and extracting categories from it. In this stage of research, a sentence-by-sentence analysis of the original materials of the interview was conducted (Tables 2 and 3).

**Table 2:** Examples of open coding process from raw data to initial categories.

Initial category	Concepts	Labelling	Raw data
Teacher’s responsibility	Moral guidance	Nurturing the whole person	I believe that the role of a teacher goes beyond just teaching—it’s also about nurturing students.
	Knowledge and skills	Physical conditioning guidance	When it comes to PE teachers, they can really make a difference in kids’ physical development, and even more so in their mental well-being.
	Improve physical fitness	Recover physical fitness	…Especially after the pandemic or outbreaks like the flu, we have seen a clear decline in students’ physical fitness. So how can we help kids recover their fitness in a way that’s both scientific and gradual…PE teachers definitely have a big part to play in that.

**Table 3:** Examples of open coding process about the initial categories.

Initial category	Concepts	Labelling
Characteristics of curriculum	Communicativeness	Communication, tolerance, intimacy, motivation, in-class communication, communication, frequent interaction
	Competitive nature	Competition, concept of winning and losing, competitive nature
	Fun	Games, fun, entertainment
	Practicality	Practice, practical courses, outdoor activities, get moving
Status of curriculum	Promotion	Receiving attention, increased proportion in exams, parental attention, national attention, status promotion, teacher status promotion
	Lack of attention	Obstruction, option for poor students, lack of support, lack of attention, class time being occupied
	Influencing factors	National curriculum, sub-health phenomenon, location, school principal, high school entrance examination, high school entrance examination scores
Functions of curriculum	Moral development	Morality, moral formation, moral education, hard work, cultivation of three outlooks
	Willpower and character	Will, endurance, sports spirit, fighting spirit, character, moral formation, sense of competition, never give up
	Sports spirit	Sports spirit, spirit cultivation
	Physical fitness	Good physique, physical fitness, physical enhancement, physical fitness, feminine culture, healthy development, health first, physical health, physical and mental development, healthy growth, physical education, physical exercise, healthy physique, physical constitution
	Knowledge and skills	Knowledge, knowledge and skills, mastery of skills, sports cognition, sensitivity, sports ability
	Mental state	More sunny, balance between work and rest, reduced depression, relaxation, stress release, relaxation, release of innate nature, physical and mental health, more outgoing, mental outlook, self-confidence, cheerful and optimistic, liberation of innate nature, balance between work and rest
	Interpersonal communication	Unity and cooperation, cooperation with others, communication skills, interpersonal communication, discipline, team collaboration, friendship promotion, cooperation, social adaptation
	Habit formation	Sports habits, enrich life, habit formation, exercise habits, get moving, healthy behaviour
	Intellectual development	Brain development, promote development, positive impact, facilitate development, intellectual education, enhance thinking, promote learning
	Aesthetic improvement	Promote development, pursuit of beauty, aesthetic improvement, sports aesthetics
	Interest cultivation	Sports hobbies, love
	Labor promotion	Labor awareness, promoting labor
	Character shaping	Character shaping
	Self-understanding	Understand sports conditions

#### Axial coding

The main purpose of axial coding is to discover and establish various connections between subcategories, thereby demonstrating the organic correlations between various parts of the data on the basis of open coding, the work of axial coding aims to further explore the logical relationships and potential connections between these initial categories, and further to develop the subcategories. In this research section, by analysing the internal connections and logical order between 36 initial categories, 10 subcategories were further extracted, namely curriculum understanding, physical education learning understanding, teacher cognition, teaching process cognition, emotional background, emotional fusion, emotional sublimation, professional behaviour experience, professional behaviour choice, and future planning (Table 4).

**Table 4:** Results of axial coding.

Subcategory	Connotation
Curriculum comprehension	Course characteristics, course status, and sports functions.
Understanding of PE learning	Student needs, student psychology, student physical condition, learning characteristics, learning attitude, and influencing factors.
Teacher cognition	Teacher qualities, teacher responsibilities, professional characteristics, and teacher image.
Cognition of the teaching process	Teaching objectives, teaching content, teaching principles, teaching behaviours, and teaching evaluation.
Initial emotions	Career expectations, self-professional evaluation, and career emotional tendencies.
Emotional development	Recognition of self-professional competence, recognition of self-vocational competence and vocational emotional experience
Emotional sublimation	The career ideals and future plan
Behavioural experience of the vocation	Teachers’ role and self-reflection
Behavioural choice of the vocation	Teaching design and implementation of PE, the approach to dealing with students and self-requirements
Future planning	Plans of teaching and plans of the vocation

#### Selective coding

Selective coding is the refinement of subcategories from the main category, and through repeated comparative analysis of the intrinsic relationships between each category, it describes the overall behavioural phenomenon in the context of a storyline. In the process of selective encoding, the author further analysed the internal connections and logical order between each initial category and subcategory, ultimately forming a three-dimensional belief system of PPETs.

### Trustworthiness and ethical considerations

The trustworthiness of the analytical process was enhanced through the implementation of multiple verification strategies. During interviews, member checks were consistently conducted by posing clarifying questions to confirm the accuracy of participant responses, and all interviewees were subsequently offered opportunities to review their transcribed accounts. To triangulate the data provided by the PPETs, follow-up interviews were carried out with their respective PE educators—including professional course instructors and academic advisors. What’s more, official curriculum documents from the three participating universities’ PETE programs were also collected. This methodological triangulation across sources ensured the credibility of student-reported information regarding courses, teaching activities, and curricular structures. Furthermore, throughout the coding phase, a reflexive research journal was maintained to support analytical transparency and methodological rigor. In the triangulation phase of this study, semi-structured interviews were conducted with teacher educators from three participating universities. The interviews cantered on the following core questions: (1) How do you perceive the role of PE teacher educators? (2) What efforts has your university undertaken in PETE programs? (3) How could your institution enhance its support for the development of PPETs?

This study was reviewed and approved by the Special Committee on Ethics of the Academic Committee of Capital University of Physical Education and Sports (Approval number: 2025A117). Informed consent was obtained from all participants prior to the interviews.

## Findings

**Figure 1:**
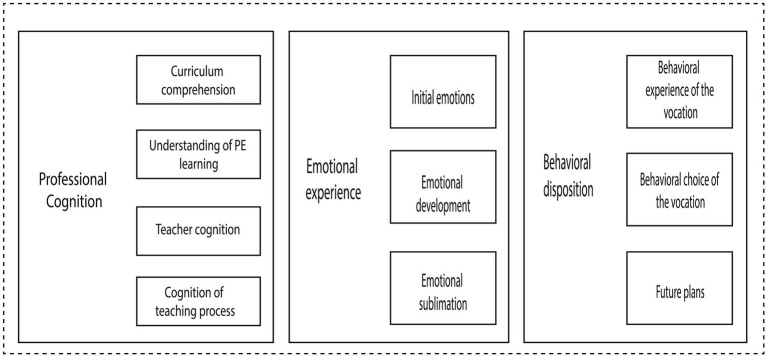
The system of PPETs’ beliefs.

Through the grounded theory, a theoretical system of PPETs belief was constructed. This system is composed of three core and interrelated dimensions: professional cognition, emotional experience, and behavioural disposition. The following sections elaborate on this structure, first detailing the composition of each dimension and then illustrating their dynamic evolution (Fig. 1).

### Dimension of professional cognition

The development of professional cognition among the PPETs in this study included four components: teacher cognition, cognition of the teaching process, curriculum comprehension, and understanding of PE learning. The interview data showed that these cognitions did not remain static. Instead, they evolved significantly from initial impressions based on personal experiences to more nuanced understandings forged through PETE courses and teaching practicum.

At the start of their university studies, the participants’ views were often broad and idealized. A common perception was that the PE teacher’s role was relatively straightforward. One participant captured this sentiment by saying, *“I used to think being a PE teacher was the easiest job—just blowing a whistle, forming lines, and then dismissing the class. It seemed like the simplest thing.”* (1-CXD).

However, as they engaged with their PETE courses and participated in simulated teaching, their thinking began to shift. Their perception of the teacher’s role transformed from seeing it as simple to recognizing its challenging and complex nature. This change is reflected in their accounts. One student shared how their perspective deepened with learning, *“The more I learned within this major, the more I realized that being a PE teacher is truly not easy. I came to understand this through personal experience.”* (1-CXD).

Their concept of a teacher’s duties also broadened. It was no longer just about teaching sports skills. As another participant explained, their view expanded to include the teacher’s holistic influence:” *Through my studies, I learned that a teacher does not only help students physically; they also provide significant support to students’ mental well-being, spirit, and behavioral habits.”* (7-CA).

A particularly interesting tension emerged around their teaching philosophy. While many entered the program with a student-centered outlook, their practicum often led them to adopt a more teacher-centered approach to establish control. This was often a conscious strategy to manage the classroom. One participant justified this shift by stating, *“I knew deeply that I could not treat them as a mere intern. Because I was independently in charge of the class, I had to use a teacher’s demeanor to restrain and manage them. I needed to regulate your words and actions in the classroom.”* (20-YD).

For some, this meant presenting a stern demeanor to preemptively secure students’ respect, a clear departure from their earlier ideals of being a friendly and respectful figure. Another participant described this strategy, *“Students seemed to think they could take advantage of me because I was a student teacher. But I did not let that happen. I was very serious with them from the beginning, wanting to establish a presence. When students talked back and did not listen to instructions, I became very strict.”* (18-ZYZ).

This contrast between their initial ideals and their practical choices in the classroom highlights the complex journey of reconciling personal beliefs with the immediate demands of teaching.

### Dimension of emotional experience

Dimension of emotional experience consists of initial emotions, emotional development and emotional sublimation. Firstly, initial emotions consist of career expectations, self-professional evaluation and career emotional tendencies. It is found that when the time PPETs registered in the university for the first time, they had beliefs which were related to affection, and that was named as initial emotions in current study. Specifically, career expectation and self-professional evaluation signified an individual’s assessment of their choice to become a PE teacher. Career emotional tendencies referred to an individual’s evaluation of the profession of PE teacher.

As one participant expressed, *“I believe the most significant concept in a person’s life is ‘legacy,’ which is immensely important. Whether it pertains to family lineage or inheritance, I consider education and teaching to be an integral part of this legacy—it is both crucial and meaningful.”* (14-YL) These statements demonstrate a strong positive professional inclination during this stage, which in turn cultivates substantial career expectations for the student. It is precisely out of the love for PE that PPETs opt for this major. It is also the emotional manifestation towards the profession of the teacher, reflecting an individual’s evaluation and attitude towards the teaching.

Secondly, emotional development spans two-time frames, namely the learning stage of PETE course during the first 3 years and the teaching practicum in the fourth year. During the first period, emotional development comprises the recognition of self-professional competence as PE-major students and recognition of self-vocational competence as PPETs. The significant feature about this emotional dimension is the shift from “Do I like it?” to “Am I capable?” For example, *“In previous PETE courses, particularly the practical sessions, when you had to conduct a teaching demonstration. I would become extremely nervous, to the point where my voice would shake. I wondered, ‘How can I become a teacher if I cannot even speak confidently in front of students?’. Personally, I felt that my demonstration of the movement was not standard enough, and my skill level had not reached the required standard. Consequently, I felt hesitant to explain the key points of the movement to students. I believed I could not teach them effectively, which resulted in a lack of self-confidence.”* (9-DX).

This account illustrates how the teaching demonstration component during PETE’s courses induced intense nervousness in the student, even causing physical symptoms like a trembling voice. Furthermore, beyond concerns about teaching skills, the student also began to doubt their own professional sports skills, believing their *“skill level had not met the standard*” (9-DX), thereby losing confidence in their professional competencies.

Thirdly, emotional sublimation refers to the professional ideals and professional concerns formulated by PPETs during the teaching practicum in the fourth year. During the third phase, PPETs predominantly reported positive emotional experiences regarding the teaching profession. For instance, one participant expressed, *“I was able to command their attention and make them genuinely enjoy physical education classes. From a teacher’s perspective, this brought a great sense of accomplishment.”* (6-XJL). Another shared, *“Seeing that students could not only enjoy my classes but also learn something from them became one of my key sources of fulfilment.”* (14-YL). As these prospective teachers entered the classroom and began to transform their understanding of both student learning and the teaching process, they came to recognize the true challenges of being a teacher. Consequently, when they successfully engaged students and facilitated tangible learning, it generated a powerful sense of professional achievement.

However, some participants also described negative emotional experiences. One stated candidly, *“I do not want to be just a teacher; my interest in teaching is not particularly strong. I aim to transition into administration eventually. As a teacher, you teach 16–17 classes a week. I might only do this for a year or two.”* (24-SGH) Another offered a more critical perspective, *“If I started out limiting myself to just being a teacher, I could essentially coast. I could make a cup of tea every day, blow the whistle a couple of times during class, and see out my career from hiring to retirement. It’s a life whose end is visible from the start.”* (21-YBQ).

These statements reveal that some PPETs hold a negative attitude towards the PE teaching career, explicitly stating their reluctance to remain in the role. The latter example, in particular, shows how their exposure during the internship to what they perceive as the uninspiring reality of a teacher’s routine—symbolized by “making a cup of tea” and “blowing the whistle a couple of times”—has fostered self-doubt about committing to a lifelong career in PE education. Through their descriptions of the status quo, it is evident that they internally view a future as a PE teacher as lacking in prospects.

### Dimension of behavioural disposition

The dimension of behavioural disposition is categorized into three components: behavioural experience of the vocation, behavioural choice of the vocation and future plans. Among them, behavioural experience of the vocation consists of the actions taken by PPETs during the period of PETE courses from the first to the third year, in activities such as simulated teaching within professional courses and informal teaching experiences in extracurricular PE practices (distinct from the formal teaching practicum in the fourth year), and these actions are manifestations of their cognition regarding PE teaching. Behavioural choice of the vocation refers to the teaching behaviours that PPETs have adopted or plan to adopt in formal school PE classes during practicum. Future plans refer to the behaviours PPETs will tend to choose a PE teacher in the future.

During the teaching practicum, PPETs’ instructional practices often manifested as a form of compromise with the practical environment, directly reflecting the internal tensions within their cognitive framework. To mitigate educational risks, they felt compelled to abandon certain aspects of their ideal instructional designs in favor of adopting a conservative, “safety-first” approach. One intern clearly articulated this compromise, *“I had originally designed a really fun game, but the moment I thought about the possibility of children falling and getting injured during the chase, I became apprehensive. In the end, I reverted to the most traditional queuing exercises. I know it’s boring, but at least nothing will go wrong.”*(5-LJ).

This excerpt vividly reveals the dilemma they faced, caught between the professional ideal of promoting physical development and the practical pressure to ensure absolute safety. Such behavioural compromises serve as external evidence of the difficult negotiation between their cognition and the real-world context.

### Division of three phases for the development of teacher beliefs

**Figure 2:**
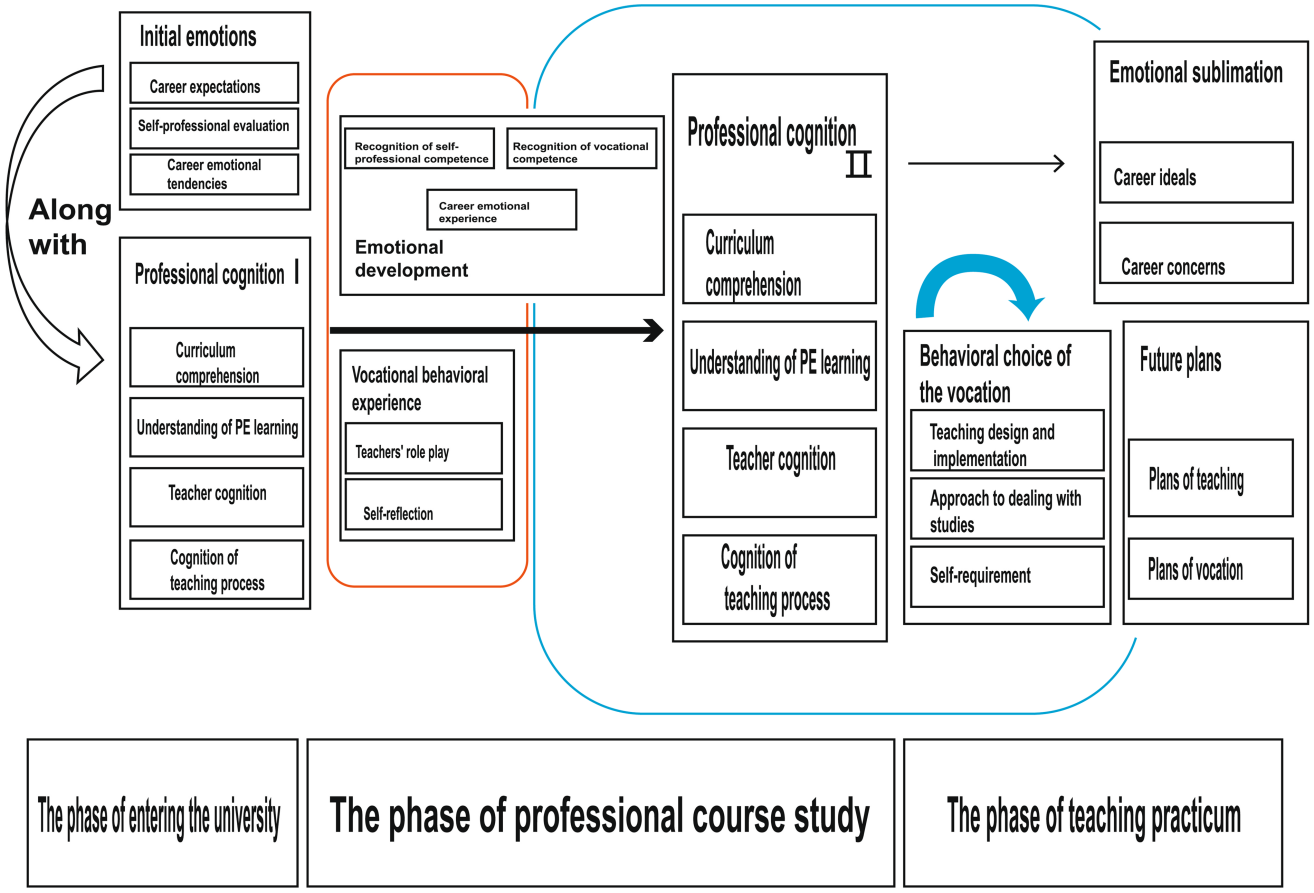
Three-phase dynamic model of PPETs beliefs development.

Based on in-depth interviews with 25 PPETs, this study proposes a three-phase dynamic model of PPETs beliefs development (Fig. 2). The research reveals that the formation of teacher beliefs follows a distinct developmental trajectory: starting from initial perceptions of the profession upon entering university, progressing through conceptual construction during PETE courses, and culminating in practical transformation during the teaching practicum. This developmental process demonstrates clear phase characteristics, with inherent connections between the three phases that build upon one another.

Specifically, PPETs’ professional cognition, emotional experiences, and behavioural disposition maintain close interactive relationships throughout their development. The deepening of professional cognition often stimulates positive emotional experiences, which in turn prompt more engaged teaching behaviours. These three dimensions form a virtuous cycle of mutual reinforcement and coordinated development.

Notably, this multidimensional interaction shows a gradual intensification throughout the 4-year university life. Particularly during the teaching practicum phase, the interplay among the three dimensions reaches its peak. In authentic teaching environments, PPETs continually need to reconcile theoretical knowledge with practical requirements, adjust their emotional attitudes, and modify their teaching behaviours.

## Discussion

This study constructed a theoretical belief system of Chinese PPETs, which is comprised of three interconnected categories: professional cognition, emotional experience, and behavioural disposition. The findings not only align with established understanding that teacher beliefs are multidimensional but further illuminate how these dimensions develop through the specific context of teacher education in China.

### The interpretation of professional cognition

This study found similarities with previous research in that PPETs talked about their understanding of PE courses, especially emphasizing the importance of PE for students’ development (Fan et al., 2018; Linker and Woods, 2018). More importantly, a key finding regarding professional cognition is that Chinese PPETs perceive their responsibilities as encompassing both the physical and the moral dimensions of education. This emphasis on the teacher’s moral role finds a strong echo in traditional Chinese educational philosophy. The classical definition of a teacher by the Tang Dynasty scholar Han Yu (768–824 CE) in his seminal essay “On Teachers” (“Shi Shuo”) defines the teacher as “one who imparts moral principles, transmits knowledge, and resolves doubts.” This definition appears to provide a cultural script that informs how these prospective teachers conceptualize their professional identity. Beyond this cultural underpinning, our data show that this cognition is not static but evolves significantly through teacher training. This observation supports Yuan (2022) view of teacher expertise as a dynamic context-responsive knowing. For example, the participants’ understanding of their role expanded from a simplistic view of a partner to include more complex identities like classroom manager and curriculum designer. This evolution was driven in the practical reality of their internship schools, a finding that lends empirical weight to Johnson and Golombek's (2020) argument for situated and goal-directed teacher education. The internship served as a critical activity system where theoretical knowledge was tested, refined, and transformed into actionable understanding.

### The interpretation of emotional experience

In terms of emotional experience, the results of this study agree with previous studies in which the concepts related to self-concept and teaching efficacy (Hand, 2014; Pendergast et al., 2011). In this study, self-profession evaluation, the recognition of self-professional competence and the recognition of self-vocational competence are related to the self-concept and teaching efficacy, which show the emotions PPETs could experience about themselves. The difference lies in that based on grounded theory, the emotional description in this study is more in details. In addition, it was also discovered that some PPETs were not initially confident in certain sports-specific abilities and the study of teacher-related courses. Instead, their confidence was developed through continuous learning, thus showing an identification with their own professional abilities.

In addition, from the data, there is an emotional trajectory of the PPETs moved from initial enthusiasm, through a phase of confidence-building and confronting setbacks, toward a more mature sense of responsibility and care. This pathway suggests that the socio-emotional capacity highlighted by Yuan (2022) is not an innate trait but is developed through key experiences. These include the affirmation gained from successful simulated teaching and the resilience forged in managing challenging classroom situations.

### The interpretation of behavioural disposition

Evidence from the studies show that teacher beliefs are related to behavioural disposition. Rokeach (1968) maintained that all beliefs, apart from encompassing cognitive components and emotional components capable of evoking emotions, also possessed a behavioural component that had activated when action was requisite.

In psychology, behavioural disposition is usually a state of psychological preparedness, that is, an individual’s preexisting inclination towards whether to engage in a certain behaviour in a specific situation. Behavioural disposition is influenced by attitudes, subjective norms, and perceived control (Ajzen, 1991). This study employs the grounded theory from the bottom-up and found that among PPETs, their behavioural disposition can refer not only to behaviours that have not actually occurred but also to behaviours that have already occurred, and the latter increases the likelihood of actual behaviour.

The behavioural disposition of the PPETs manifested as a sequence from simulated teaching, to reflective decision-making in real internships, and finally to concrete future planning. This pattern aligns with Fonseca-Chacana (2019) concept of dispositions as properties that guide wise practical action. A clear example was how some participants, who began with a strictly student-centered approach, learned to integrate necessary rule-setting after experiencing classroom management issues, demonstrating a context-sensitive adjustment of their practice.

### Some suggestions for PETE programs

According to the research, there are generally two suggestions for PE teacher educators. Firstly, during the 4-year PETE programs, teacher educators should systematically create contextualized teaching environments. These environments, such as simulated teaching sessions or micro-lessons within PETE courses, provide platforms for PPETs to engage in practical teaching experiences. Through such role-play, PPETs transform from knowledge and skill receivers into knowledge and skill facilitators, leading to cognitive shifts in their understanding of teaching processes and learning. Secondly, negative emotional experiences during teaching practicum can weaken their professional beliefs. Therefore, it is essential for supervisors of both universities and intern schools to closely monitor and support the emotional well-being of PPETs. This includes regularly listening to their reflections, addressing feelings of burnout or disappointment, providing constructive feedback, and guiding them in self-evaluation and professional goal-setting based on self-awareness.

### Limitation

This study has some limitations. Grounded theory coding, as a relatively scientific and mature approach, although it possesses explicit coding processes and norms, is still in its nascent stage in the domain of sports studies in China, and the standardization employed in research requires further exploration. Furthermore, the grounded theory coding process poses considerable demands on the researcher’s research capabilities. As a research instrument, the researcher is obliged to have a superior level of abstract generalization ability, deductive reasoning capacity, and theoretical sensitivity. During the research process, although the researcher has engaged in relevant learning and extensive reading regarding the research methods of grounded theory, numerous perplexities still emerged in the actual operation, and continuous learning is necessary to enhance the research quality of this method.

## Conclusion

This research examines the system of teacher beliefs of PPETs in China. Firstly, based on the grounded theory, it is found that the system of teacher beliefs of PPETs has three core categories including professional cognition, emotional experience and behavioural disposition. Furthermore, the features of the system suggest that the forming process of PPETs’ belief during PETE programs is dynamic. With the findings, our study makes several theoretical and practical contributions.

The current study supplemented the gap in the system of teacher beliefs, refined the emotional and behavioural elements in the system based on interview data, and discovered that during the 4-year university period, the system of teacher beliefs of PPETs presented a dynamic development.

## Data Availability

The original contributions presented in the study are included in the article/supplementary material, further inquiries can be directed to the corresponding author.
